# Homonymous hemianopia in childhood: a systematic scoping review protocol

**DOI:** 10.1136/bmjophth-2022-001073

**Published:** 2022-12-01

**Authors:** Sian Handley, Richard Bowman, Alki Liasis, Jugnoo Sangeeta Rahi, the Homonymous Hemianopia in Childhood study group

**Affiliations:** 1 Ulverscroft Vision Research Group, Great Ormond Street Institute of Child Health, UCL, London, UK; 2 Clinical and Academic Department of Ophthalmology, Great Ormond Street Hospital for Children, London, UK; 3 Population, Policy & Practice Dept, Great Ormond Street Institute of Child Health, UCL, London, UK; 4 School of Medicine, University of Pittsburgh, Pittsburgh, Pennsylvania, USA; 5 Department of Ophthalmology, Children's Hospital of Pittsburgh, University of Pittsburgh Medical Center Health System, Pittsburgh, Pennsylvania, USA; 6 NIHR Moorfields Biomedical Research Centre, London, UK; 7 Institute of Ophthalmology, University College London, London, UK

**Keywords:** child health (paediatrics), field of vision, visual (cerebral) cortex, visual pathway

## Abstract

**Introduction:**

Cerebral visual impairment (CVI) is the leading cause of visual impairment in childhood in western countries. This encompasses a heterogeneous group of disorders and a spectrum of types of visual impairments. Research is required to explore specific subtypes of CVI and the specific needs of these groups to provide more individualised patient care. One type of CVI is homonymous hemianopia (HH), the absence of vision on one side due to an insult to the postchiasmal visual pathways in one hemisphere of the brain. The scoping review aims to map the nature, features and volume of the existing literature around HH in infancy, childhood and young adolescence.

**Methods and analysis:**

We will perform a scoping review of the literature relating to HH in children (0–18 years old). The review will follow the PRISMA extension for scoping reviews checklist to ensure reporting integrity. We will conduct electronic database searches including CENTRAL, CINAHL, MEDLINE (PubMed) and PsycINFO. We will also carry out a ‘grey literature’ and internet search for studies or materials not formally published. Two researchers will independently review abstracts yielded from the search strategy for study inclusion.

**Dissemination:**

This review will inform health professionals and other stakeholders working within this growing population of children with CVI. Our review will summarise the literature relating to this specific subgroup of CVI, and will identify gaps that require further research and development towards specific care of children with this form of CVI.

**WHAT IS ALREADY KNOWN ON THIS TOPIC**

- Cerebral visual impairment (CVI) is the leading cause of childhood visual impairment in western counties with incidence increasing. Homonymous hemianopia is a specific subtype of CVI.

**WHAT THIS STUDY ADDS**

- The scoping review protocol documents the study methods to explore the nature, features and volume of the existing literature around homonymous hemianopia in children.

**HOW THIS STUDY MIGHT AFFECT RESEARCH, PRACTICE OR POLICY**

- Mapping the current literature specific to homonymous hemianopia in children will provide a synthesis of the information to provide a resource for clinicians, carers, patients and the public while identifying areas of paucity requiring future research. If the scoping review proves beneficial to the relevant stakeholders the methodology could be used to scope the literature in other specific subtypes of CVI.

## Background and rationale

Cerebral visual impairment (CVI) is the leading cause of visual impairment in children in developed countries.^1 2^ It is defined as a verifiable visual dysfunction, which cannot be attributed to disorders of the anterior visual pathways or any potentially co-occurring ocular condition.^3^ The heterogeneous nature of CVI means that in clinical practice children present with a wide spectrum of visual deficits and associated challenges faced by both the child and their carers. Therefore, it is becoming acknowledged that a ‘one-size-fits-all’ approach to CVI is not appropriate, with an increasing call for an adaptive approach using evidence-based knowledge tailored to give individualised care to the child with CVI.^3–6^

Homonymous hemianopia (HH) is one specific type of CVI seen in children. HH is the loss or absence of one half of the visual field affecting the same side in both eyes. It is caused by damage to the retrochiasmal visual pathways of one side (hemisphere) of the brain, with causes reported in children including brain injury, stroke or tumour.^7–9^

There are potential barriers when searching for published information about HH in childhood. First, it is relatively rare in children compared with in adult populations, with children representing just 9.5% of a published cohort of patients with HH.^7^ Therefore, the published literature is likely to be heavily dominated by studies in adults. Second information about HH in childhood (as well as other specific types of CVI) might be within studies that encompass the wider CVI spectrum making dissemination potentially more challenging.

To investigate this, a scoping review will be developed and undertaken to map, summarise and disseminate the existing research on HH in childhood.

The scoping review will address the following objectives:

1. To map the nature, features and volume of the existing evidence/literature around HH in infancy/childhood/young adolescence (age 0–18).
2. To summarise and disseminate the existing research.
3. To identify gaps to make recommendations for future work.
4. To collate any freely available grey literature resources (ie, patient information leaflets or websites) that may support stakeholders such as children with HH, their families or carers, teachers, doctors and allied health professionals.

## Methods

The scoping review protocol development followed frameworks by Arksey and O’Malley, Levac *et al* and the Joanna Briggs Institute guidance.^10–13^ The Preferred Reporting Items for Systematic Reviews and Meta-Analyses (PRISMA) extension for Scoping Reviews (PRISMAScR) checklist was used to ensure the reporting of the protocol and integrity of the study design.^14^

### Inclusion and exclusion criteria

The inclusion and exclusion criteria were designed by the study team to capture the full breadth of literature on the topic. The criteria are as follows:

1. Include primary research studies published in peer-review journals.
2. Given the rarity of the condition-case reports and conference, abstracts will be accepted as long as they have been peer-reviewed.
3. Include grey literature from grey literature databases searches.
4. Language: English.
5. Exclude animal studies.
6. Exclude studies with genetic or laboratory findings only, that is without any clinical data.
7. Include websites of organisations/bodies relevant to the patient population (ie, charities, hospitals, visual impairment schools, conference organisers, universities) found via Google. Exclude other Internet sources from the Google search.
8. Exclude other forms of hemianopia, that is, bitemporal, binasal, superior, inferior or monocular.

### Search strategy

Keywords concerning the target conditions and visual fields will be used, these are summarised in table 1. A separate MeSH terms search (table 2) will be run in PubMed (MEDLINE) in addition to the keyword search. The field code [all fields] will be used and all MeSH terms exploded in the search process.

**Table 1:** Planned search strategy—keywords to be searched with Boolean operators

	AND	
**OR**	1)Infan*	16)Hemianopsia, Homonymous
	2)Newborn*	17)Hemianopsias, Homonymous
	3)Baby*	18)Hemianopia, Homonymous
	4)Babies	19)Hemianopias, Homonymous
	5)Neonat*	20)Homonymous Hemianopsia
	6)Child*	21)Homonymous Hemianopsias
	7)Schoolchild*	22)Homonymous Hemianopia
	8)Preschool*	23)Homonymous Hemianopias
	9)Toddler*	24)Hemianopia
	10)Teen*	25)Hemianopsia
	11)Adolesc*	
	12)Pediatric*	
	13)Paediatric*	
	14)Congenital	
	15)1 OR 2 OR 3 OR 4 OR 5 OR 6 OR 7 OR 8 OR 9 OR 10 OR 11 OR 12 OR 13 OR 14	26)16 OR 17 OR 18 OR 19 OR 20 OR 21 OR 22 OR 23 OR 24 OR 25
27)15 AND 26		

Keywords will be entered in the order presented. In step 15 Keywords 1–14 will be combined with the OR operator, and in step 26 keywords 16–25 arecombined with the OR operator. In the final step 27, steps 15 and 26 are combined with the AND operator.

**Table 2:** MeSH term search for PubMed

	AND	
OR	1)Infant [Mesh]	5)Hemianopsia [Mesh]
	2)Child [Mesh]	
	3)Adolescent [Mesh]	
	4)1 OR 2 OR 3	
	6)4 AND 5	

Each term is labelled in order it will be entered. In step 4 the MeSH terms 1–3 are combined with the OR operative. In step 6, steps 4 and 5 are combined with the operator AND.

### Information sources for search

#### Database search

A systematic strategy to search key electronic databases, including Cochrane registers and electronic bibliographic databases will be used. This also includes smaller ophthalmology-based databases. All databases will be search in their full date range until of search. The date range searched will be reported for each database in the scoping review. The databases to be searched are as follows:

1. AMED.
2. British Nursing Index.
3. CINAHL.
4. ClinicalTrials.gov.
5. Cochrane Central Register of Controlled Trials (CENTRAL).
6. Cochrane database of systematic reviews.
7. Cochrane Eyes and Vision Group Trials Register.
8. Current Controlled Trials.
9. Health Service Research Projects in Progress.
10. National Eye Institute Clinical Studies Database.
11. Orthoptic Search Facility.
12. Proceedings of Association for Research in Vision and Ophthalmology.
13. PsycBITE (Psychological Database for Brain Impairment Treatment Efficacy).
14. PsycINFO.
15. PubMed (MEDLINE).
16. Trials Central.

#### Grey literature search

A search of additional grey literature databases will also be conducted to capture additional information that may not be present in the key electronic registers and databases but still valuable sources of information such as patient information leaflets. The following grey literature sources will be searched using “homonymous hemianopia” unless otherwise specified and then results in hand refined for those relating to children. If this provides too many results the study team and senior author will be consulted before changing the search terms. The grey literature databases that will be searched are:

1. PsycEXTRA.
2. Open Grey.
3. HMIC - Health Management Information Centre.
4. Open DOAR.
5. TRIP medical database.
6. PsyArXiv
7. medRxiv.

#### Internet search

Additional grey literature will be searched through the internet search engine Google. Searching the internet could lead us open to identifying information that is not robust, an individual’s extreme opinion or indeed completely false. Accidentally disseminating inaccurate information in the scoping review could be potentially detrimental to the stakeholders and care providers this review aims to support. However, the risk of this has to be weighed up against the potentially valuable information the Internet also houses. For this review, we propose to identify and include web pages from organisations who are accountable for the quality of the information on their website. We suggest that because of this accountability it is likely of reasonable quality and that it is also likely the information has been internally reviewed before publication. Examples of these are registered charities, visual impairment schools, medical research conferences and university or hospital websites. The Google search “Homonymous Hemianopia in children” will be used. Given the potential breadth of results that could come from the internet and the potential ambiguity of the definition of includable sources, the search will be separately run by two reviewers. They will meet to compare search results and consensus required before inclusion of any webpages.

### Study selection and management

Records from all databases will be exported into and managed in Covidence systematic review software (Veritas Health Innovation, Melbourne, Australia, available at www.covidence.org). The search and eligibility screening findings will be reported both as a narrative description and by populating the PRISMA-ScR flow diagram to provide a visual representation of the data selection process.^14^

The records will be searched for duplicates, and duplicate studies will be removed. Two researchers will independently review abstracts yielded from the search strategy for study inclusion.

The reviewers will meet at the beginning, midpoint and final stages of the abstract review process to discuss any challenges or uncertainties, and refine the search strategy if needed. In the event of any disagreement between the two reviewers, the senior author will hold the deciding vote.

Full data extraction for charting will be carried out by the first reviewer. A second reviewer will independently extract the full data from a random sample of 33% of eligible literature. If there is more than 10% disagreement, the senior author will be consulted and the second reviewer will extract the full data set for all eligible literature.

### Charting the data

The study team has collectively developed the data charting form. Two reviewers will independently pilot chart the first 5–10 studies and then meet to discuss consistency and/or any problems found. Any unresolved issues would be directed to the senior investigator and study group for further advice. The charting form has been designed to capture the following fields:

1. Biometrics (authors, publication year, title and journal or source).
2. Type of study (or type of grey literature).
3. Country of origin.
4. Aims/purpose.
5. Sample size.
6. Paediatric age range.
7. Cause of HH in the sample.
8. Intervention and comparator (if applicable).
9. Duration of intervention (if applicable).
10. How outcomes are measured.
11. Key findings that relate to review question.
12. Evidence level
13. Classification of Key Topic from these following topics:
  - Incidence of HH.
  - Causative pathology of HH.
  - Diagnosis of HH.
  - Natural history/progression/adaptation of HH.
  - Standard/non-experimental ophthalmological management of HH.
  - Interventions for HH.
  - Patient advisory, patient education or support information.

### Result reporting

The data from the charting will be mapped in different formats to meet the aims of the scoping review. As the protocol is being developed ahead of undertaking the scoping review, it is not possible to know the volume and nature of literature that will be charted and therefore we cannot be conclusive about the best mapping methods. It is also possible that additional ideas for mapping might develop while the literature is being searched or charted. Any potential new maps or map changes would be discussed with the senior author before implementation. Any mapping outside of the protocol would be justified in the reporting of the scoping review.

The proposed mapping methods would include:

- A graph would be used to map the evidence levels of the literature found (y) against the date of publication (x). The individual points will be colour coded to reflect key topic.
- A proportional symbol map will be used to show the world distribution of the origin of the literature found. Depending on the nature and extent of literature found, bubbles could be broken down into pie charts to display the literature key topic, including a pie chart for the total literature for comparison.
- The reported causes of HH in cohorts of children in different literature sources could be displayed on a proportional symbol map with proportions of pathology displayed in pie charts, including one to summarise all studies. Each pie chart would be annotated with the number of cases.
- The nature and location of patient and family information grey literature sources that are freely available would be put into a table.

### Patient and public involvement

Children with HH and their parents/carers were involved throughout the design of the Homonymous Hemianopia in Childhood project. When asked about their research priorities many parents and/or carers identified difficulties in accessing information about HH specific to infants and children. This led to the development of the scoping review. The children and their parents/carers will be invited to participate in the optional stage 6 consultation.^13^ The purpose of this would be to share the preliminary scoping study findings and develop effective dissemination strategies.

## Discussion and conclusion

We plan to scope the extent and nature of literature relating to HH in children to provide a unique summary of current knowledge of this subtype of CVI and to provide a single evidence-based resource that could be beneficial to inform current patient care. As well as synthesising the available literature, will in turn identify the gaps or areas of minimal research. Detection of these gaps will highlight areas requiring further research to drive forward developments in areas such as diagnosis, patient care and (re)habilitation.

As well as its potential use in clinical care and research, the mapped evidence base could have a role in public and patient involvement or stakeholder engagement. This is a suggested additional step to the scoping review process.^12 13^ The visual maps of the data could be used to create or further develop quality patient education and information resources.

If this literature synthesis proves beneficial to care professionals and other stakeholders, this methodology could be used to scope the literature in other specific subtypes of CVI. This will add to the evidence to inform more individualised patient care and show areas needed for development in this and other CVI subgroups.

## Data Availability

All data relevant to the study are included in the article or uploaded as online supplemental information.
